# Morphologic assessment of photoreceptors after idiopathic epiretinal membrane surgery with adaptive-optics SLO

**DOI:** 10.1186/s12886-026-04681-4

**Published:** 2026-02-21

**Authors:** Pengfeng Li, Yijing Zhuang, Dong Fang, Jia Liang, Yingying Diao, Huiyan Zheng, Lu Chen, Shaochong Zhang

**Affiliations:** Shenzhen Key Ophthalmic Laboratory, Shenzhen Eye Hospital, Jinan University, 18 Zetian Road, Shenzhen, Guangdong 518040 China

**Keywords:** Idiopathic epiretinal membrane, Adaptive optics scanning light ophthalmoscopy, Photoreceptors, Optical coherence tomography, Optical coherence tomography angiography

## Abstract

**Purpose:**

This study aims to quantitatively assess morphological alterations in cone cells following idiopathic epiretinal membrane (IERM) surgery using adaptive optics scanning light ophthalmoscopy (AO-SLO) and to correlate these findings with retinal structure, retina vascular characteristic and visual function.

**Method:**

This cross-sectional analysis involved seventeen eyes from 17 patients who underwent IERM surgery, compared to a control group of 35 demographically matched subjects. Comprehensive ophthalmologic evaluations were performed, including AO-SLO, Optical coherence tomography (OCT), and Optical coherence tomography angiography (OCTA). The study focused on assessing post-surgery cone density, dispersion, and regularity, as well as their relationship with visual function, macular structure, and macular vessel characteristics.

**Result:**

A significant reduction in cone densities and regularity, along with a substantial increase in cone dispersion, were observed in all quadrants of eyes after IERM surgery compared to healthy eyes (mean cone densities: t=-4.76; *P* < 0.001; mean cone regularity: t=-5.11, *P* < 0.001; mean cone dispersion: t = 5.56, *P* < 0.001). Negative correlations were found between cone densities and best-corrected visual acuity (BCVA) (ρ=-0.62, *P* < 0.001). Additionally, average cone density correlated negatively with inner retinal thickness, particularly in the inferior quadrants (ρ = -0.42, *P* = 0.03), and with vascular density of the superficial capillary plexus (SCP) in the fovea (ρ = -0.39, *P* = 0.04), as indicated by OCTA.

**Conclusion:**

The assessments of cone morphology using AO-SLO demonstrated persistent deviations from typical photoreceptor arrangements after IERM surgery, which are significantly associated with incomplete recovery of visual function. AO-SLO serves as an effective quantitative tool for identifying photoreceptor abnormalities in eyes after IERM surgery.

## Introduction

Idiopathic epiretinal membrane (IERM), characterised by the development of a nonvascular fibrocellular proliferation on the surface of the internal limiting membrane, is a prevalent retinal condition in China, with a prevalence rate of 13%, which increases with age [1, 2]. The traction exerted by IERM often results in considerable disruption of macular structure, leading to moderate to severe visual impairment in the majority of IERM patients [3]. While epiretinal membrane surgery is effective in alleviating symptoms and partially improving visual acuity, a significant proportion of patients experience incomplete visual function recovery, with some even reporting postoperative deterioration in vision [4]. The recovery of visual function is closely linked not only to the technical success of the surgery but also to the extent of retinal structural restoration and the morphological changes in retinal cells. Cone cells in the central retina play a crucial role in determining visual acuity, color perception, and overall retinal function.

With the introduction of Optical coherence tomography (OCT), clinicians can evaluate the microstructure alterations in the macular region with qualitative and quantitative precision [5]. Currently, OCT-based studies have demonstrated that structural alterations within the photoreceptor layer, such as the disruption of the photoreceptor inner and outer segment junction, and the defection of cone outer segment tips, are significantly correlated with visual acuity in eyes with IERM [6, 7]. However, conventional OCT technology often falls short in delineating the morphological alterations of foveal photoreceptor cells with sufficient clarity, despite their critical importance for assessing visual prognosis in IERM [8]. 

With advancements in optical imaging technology, adaptive optics scanning light ophthalmoscopy (AO-SLO) has emerged as a high-resolution retinal imaging tool capable of quantitatively evaluating the morphology of photoreceptor cells [9]. AO technology consists of a wavefront sensor for measuring the aberrations on the eye’s surface and a deformable mirror or a spatial light modulator that adjusts to compensate for these aberrations in real-time with living eyes [10]. By integrating Adaptive Optics technology into scanning light ophthalmoscopy, AO-SLO can offer micron-level resolution, enabling precise observation of photoreceptor cell morphology and providing valuable insights into the structural changes following retinal diseases [11]. 

This study aims to quantitatively analyze the morphology of cone photoreceptors in patients treated with IERM surgery using AO-SLO technology. By correlating these morphological changes with postoperative retinal structure, retinal vascular features, and visual function, and by comparing them to a healthy control group, we aim to reveal the density, distribution, and regularity of cone photoreceptors after IERM surgery. Moreover, we aim to explore their association with the best-corrected visual acuity (BCVA), macular structure, and retinal vascular characteristics. This research offers novel insights into the intricate relationship between cone photoreceptor morphological changes and visual function recovery after IERM surgery. Furthermore, it offers a more precise imaging tool for the clinical evaluation of postoperative visual recovery in patients.

## Method

This cross-sectional study was approved by the Institutional Review Board of Shenzhen Eye Hospital (Shenzhen China, Ethical Approval Number: 2023KYPJ093) and was conducted in accordance with the World Medical Association Declaration of Helsinki.

### Participants

Seventeen eyes of 17 patients with unilateral IERM who had undergone pars plana vitrectomy and 35 control subjects with a similar age range and gender ratio were enrolled in the study. The surgeries were performed at Shenzhen Eye Hospital by experienced vitreoretinal surgeons from October 2021 through December 2022. The surgery was performed using a 27G three-port incision, during which the vitreous was removed, and the macular epiretinal membrane along with the internal limiting membrane was peeled. No intraocular gas or silicone oil tamponade was used in any patient. In some cases, cataract surgery was performed concurrently with vitrectomy and membrane peeling.

All participants underwent a comprehensive ophthalmologic examination, including BCVA, axial length (AL) measurement using the IOL Master (Carl Zeiss Meditec, Jena, Germany), microperimetry (MP-3, NIDEK Technologies, Japan), OCT (RTVue-XR Avanti; Optovue, Inc., Fremont, CA, USA), OCT angiography (OCTA, RTVue-XR Avanti; Optovue, Inc., Fremont, CA, USA) and AO-SLO imaging system (Mona II, Robotrak Technologies, Nanjing, China).

To guarantee the integrity of image quality and to ensure the accuracy of the results, participants were excluded from the study if their eyes met any of the following criteria: (1) history of prior eye surgery except for surgery related to IERM and cataract surgery; (2) the presence of any other eye conditions or opacities that could potentially affect the visualization or morphology of the cones, such as severe cataracts, inherited retinal disorders, maculopathy, and diabetic retinopathy; (3) previous ocular injuries; (4) insufficient pupil dilation(< 3 mm), significant irregularity in pupil shape; (5) an inability to maintain stable fixation; (6) Patients with diabetes mellitus, systemic diseases that may cause retinal degeneration, or those receiving medications known to affect macular function (e.g., hydroxychloroquine). Additionally, any imaging of poor quality was excluded from the study.

### AO-SLO

AO images were obtained from a commercialized AO-SLO system (Mona II, Robotrak Technologies, Nanjing, China). This system utilizes an 840 nm light source with a full-width half-maximum (FWHM) of 40 nm. Horizontal scanning is accomplished using an 8 kHz resonant scanner mirror, while vertical scanning is achieved with a 14 Hz galvanometer mirror, yielding a 14 Hz frame rate. To maintain confocality, a pinhole with a diameter equal to approximately 2 Airy disks is positioned in front of an Avalanche Photo Diode (APD) detector [12]. For the accurate correction of ocular aberrations, the system incorporates a high-speed deformable mirror, which operates in conjunction with a custom Shack-Hartmann wavefront sensor. This synergistic arrangement ensures precise aberration correction, enhancing the imaging quality of the AO-SLO system. To safeguard the subject’s ocular health, the imaging power entering the subject’s pupil is meticulously controlled, remaining below 600 µW and adheres to the stringent safety standard defined by American National Standards Institute (ANSI). The system offers three modes for capturing retinal images, each corresponding to different field of view on the retina of 5 × 5° (approximately 1500 × 1500 μm), 2.4 × 2.4° (approximately 700 × 700 μm), 1.2 × 1.2° (approximately 350 × 350 μm). Prior to image acquisition, previously measured axial length and refractive parameters were entered into the AO-SLO imaging system to perform lateral scaling correction of individual retinal images, thereby accounting for inter-individual differences in retinal magnification. Subsequently, pharmacological pupil dilation was achieved using compound tropicamide eye drops (each 1 mL containing 5 mg of tropicamide and 5 mg of phenylephrine hydrochloride). During imaging, participants were instructed to fixate on an operator-controlled green target, and retinal images were acquired at a retinal eccentricity of 3°.

**Fig. 1:**
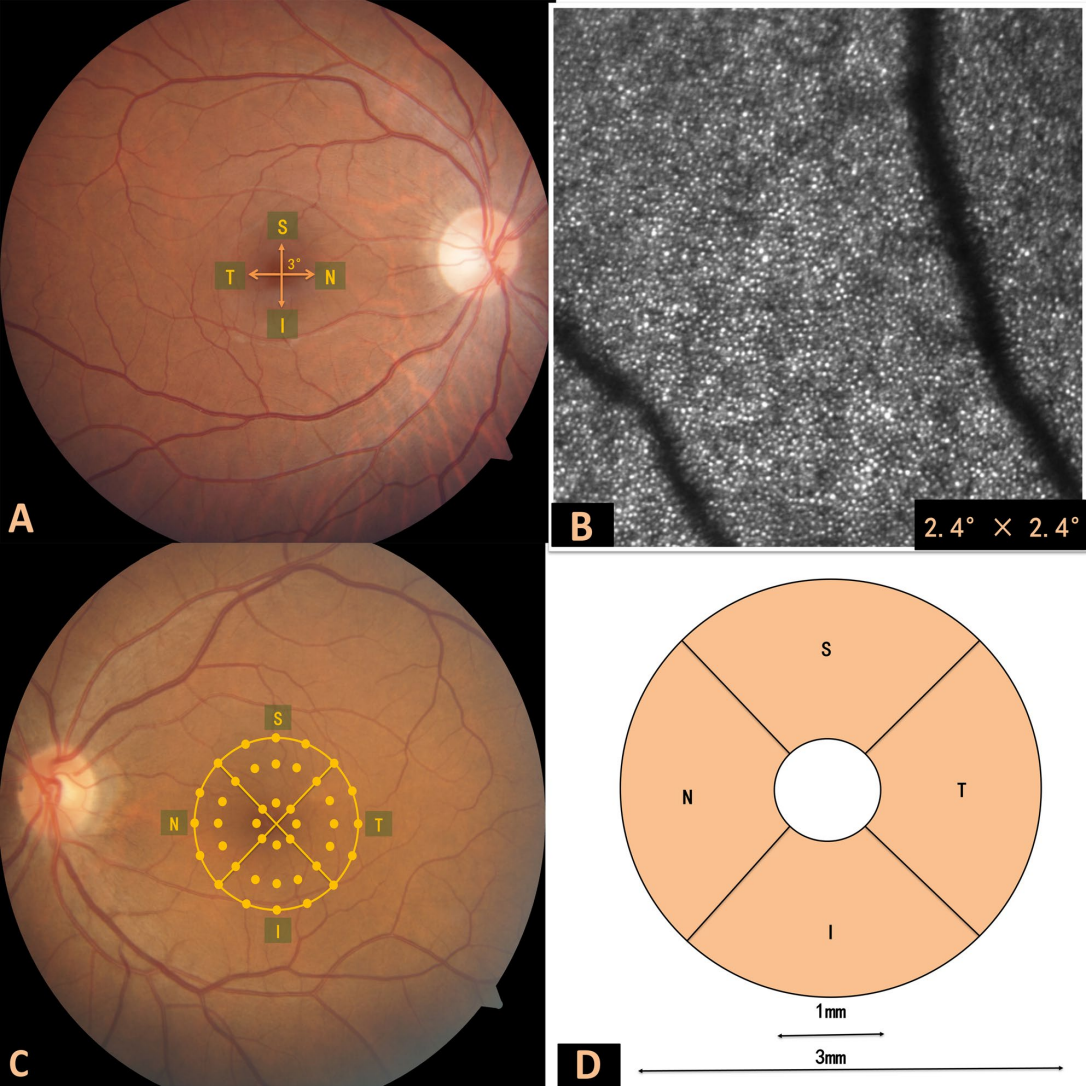
Analysis regions of a normal eye for cone morphology analysis by AO-SLO. (**A**) Capturing AO images at 3°of eccentricity from the fovea, covering the superior and inferior quadrants along the vertical axis, and the nasal and temporal quadrants along the horizontal axis; (**B**) The assessment of cone metrics is performed in the retinal view field of 2.4 × 2.4°; (**C**) The microperimetry test is conducted within a 5° (approximately 1500 μm) eccentricity region centered around the fovea of the macula. The mean retinal sensitivity (MS) is divided into four quadrants—superior, inferior, nasal, and temporal—by two diagonal lines oriented at 45° and 135°. The MS on these diagonal lines is calculated by shifting from the nasal to the superior quadrant and then to the next quadrant; (**D**) The superficial capillary density is segmented by the system’s built-in software into two regions: the foveal region with a radius of 500 μm centered on the fovea and the annular parafoveal region extending from 500 μm to 1500 μm from the fovea. The parafoveal region is further divided into four quadrants—superior, inferior, nasal, and temporal—using diagonal lines oriented at 45° and 135°

Images with substandard quality were excluded from the analysis to ensure the reliability and validity of the results of the study. For quantitative cell analysis, the apparatus is equipped with software that employs an artificial intelligence-based algorithm for automatic cone segmentation and generates statistical descriptors of cone morphology properties including density, dispersion and regularity [13]. Cone density is defined as the number of cones per square millimeter. Cone cell dispersion is the ratio of the average distance between a cell and its nearest neighbor to the variance of these distances, thereby quantifying the extent of cell dispersion or aggregation. Cone cell regularity is determined by the proportion of cells with a specific number of nearest neighbors within a defined distance, indicating the uniformity of cell distribution. A regularity value approaching 6 typically signifies a more uniform cell arrangement. To better estimate cone morphology, the retina was subdivided into four regions at 3° eccentricity: superior, inferior, nasal and temporal (Fig. 1A). The study intentionally excluded the assessment of cone morphology at the foveal center owing to the limitations of the system’s resolution. The local cone morphology was analyzed at 3°eccentricity along the four quadrants in the imaging field at 2.4 × 2.4° (Fig. 1B).

### OCT

OCT images were captured using a spectral-domain OCT (RTVue-XR Avanti; Optovue, Inc., Fremont, CA, USA) by an experienced examiner. Each eye underwent vertical, horizontal, and radial OCT scans (B-scan length: 6 mm) intersecting the center of the fovea. Using the built-in caliper tool of the software, measurements of retinal thickness (RT), inner retinal thickness (from the vitreoretinal interface to the outer border of the inner plexiform layer), outer retinal thickness (from the outer border of the inner plexiform layer to the outer border of the retinal pigment epithelium), and choroidal thickness were obtained at an eccentricity of 0°. The averages of these measurements from both the vertical and horizontal OCT images were calculated.

### OCTA

All participants underwent macular (3 × 3 mm) OCTA scans using Spectral Domain OCT (Carl Zeiss Meditec, Inc.) with Angio Vue software. This software evaluated vascular densities (VD) of superficial capillary plexuses (SCPs) and the foveal avascular zone (FAZ), segmenting the SCP into a 1–3 mm ring around the foveal center and dividing it into four quadrants: superior, inferior, nasal, and temporal (Fig. 1D).

### Microperimetry

All participants underwent mean retinal sensitivity (MS) analysis using microperimetry (MP-3, NIDEK Technologies, Japan) under dark-adapted conditions with pharmacologically dilated pupils. An assessment of retinal sensitivity thresholds was conducted within a 5° diameter around the macular centre of the retina (1° = 300 micrometers; hence 5°=1500 micrometers). The MS data were segmented into four quadrants—superior, inferior, nasal, and temporal—for analysis, with these quadrants being defined by 45° and 135° angular orientations relative to the central macular region (Fig. 1C).

### Statistical analysis

Data analyses were performed using SPSS version 27 for Windows (SPSS Inc., IBM, Chicago, IL, USA). Descriptive statistics were used to compare demographic characteristics between groups (sex, age, spherical equivalent, axial length, BCVA and MS). The normality of the data distribution was confirmed using the Shapiro–Wilks test. Continuous variables between the IERM surgery group and the control group were compared using independent sample t-tests. Pearson correlation analysis was employed to evaluate the relationships among cone morphology, OCT and OCTA characteristics, and visual function. Statistical significance was defined as a P-value less than 0.05.

## Result

### Demographic and clinical characteristics

Seventeen eyes from 17 patients(6 female, 11 male)with unilateral IERM post-surgery were enrolled. As a control group, 35 eyes from 35 subjects with comparable age distribution and sex ratio were recruited, with no or unilateral ocular disease (19 female, 16 male). Only healthy eyes were included in the control group for the study. In all surgical cases, the IERM and the internal limiting membrane were successfully peeled from the affected eyes of unilateral IERM patients without any significant postoperative complications, such as retinal detachment, choroidal detachment and infection. The mean postoperative time in the IERM surgery group was 18.06 months. There were no significant differences in the spherical equivalent (SE) and AL (SE: -0.88 ± 1.97 D vs. -0.40 ± 2.30 D, *t* = -0.66, *P* = 0.52; AL: 23.74 ± 1.15 mm vs. 23.69 ± 1.13 mm, *t* = 0.12, *P* = 0.91) between the IERM surgery group and the control group. However, a significant difference in BCVA and MS was noted between the groups (BCVA: 0.20 ± 0.16 vs. 0.02 ± 0.04, t = 4.72, *P*<0.001; MS: 24.96 ± 3.14dB vs. 27.55 ± 1.41dB, t = -2.78, *P* = 0.01). Additionally, microperimetry subfield analysis revealed statistically significant differences in MS across the inferior, nasal, and temporal quadrants of the retina when comparing the two groups (all *P* < 0.05). The demographic and clinical characteristics of the participants in both groups are presented in Table 1.

**Table 1:** Patient characteristics of the idiopathic epiretinal membrane (IERM) surgery group and the control group

	IERM surgery group (*n* = 17)	Control group (*n* = 35)	t	P value
Sex				
Female Male	11 6	19 16	— —	— —
Age (years)(range)	56.12 ± 10.48	50.86 ± 11.94	1.55	0.128
Best corrected visual acuity (logMAR)	0.20 ± 0.16	0.02 ± 0.04	4.72	<0.001***
Retinal sensitivity (Mean) (dB)	24.96 ± 3.14	27.55 ± 1.41	-2.78	<0.01*
Superior Inferior Nasal Temporal	25.61 ± 3.15 24.39 ± 4.52 24.81 ± 4.50 25.09 ± 2.78	27.13 ± 1.75 27.62 ± 1.37 27.78 ± 1.58 27.70 ± 1.33	-1.55 -2.37 -2.17 -3.12	0.14 0.03* 0.04* <0.01**
Spherical equivalent (D)	-0.88 ± 1.97	-0.40 ± 2.30	-0.66	0.52
Axial Length (mm)	23.74 ± 1.15	23.69 ± 1.13	0.12	0.91

*P＜0.05,  **P＜0.01,  ***P＜0.001

Data are expressed as mean ± standard deviation. Differences between the two groups were assessed using the independent samples t-test. t, t value of the independent samples t-test

### OCT measurement

**Fig. 2:**
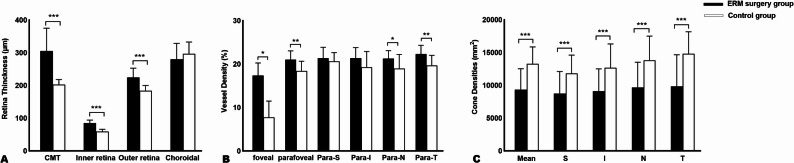
Bar graphs showing the retina structure, retina vessel characteristic and cone densities of the IERM surgery group and the control group; (**A**) CMT, inner and outer retinal thickness are significantly increased, and choroidal thickness is slightly but not significantly, decreased compared to the control group; (**B**) VD according to 3 mm automatic sectors in the SCP of the IERM surgery group and the control group is shown as percent VD. The VD is significantly increased in the fovea, parafovea, nasal, and temporal sectors in the IERM surgery group compared to the control group; (**C**) In the IERM surgery group, cone densities were significantly decreased compared to the control group, both on average and in all four quadrants. Abbreviations: IERM, Idiopathic epiretinal membrane; CMT, Central Macular Thickness; VD, Vessel density; SCP, Superficial capillary plexus

Macular structure analysis, as assessed by OCT, demonstrated significantly thicker central macula in the IERM surgery group compared to the control group (305.47 ± 69.67 μm vs. 204.67 ± 17.40 μm, t = 5.58, *P*<0.001), along with increased inner retinal thickness (85.07 ± 9.26 μm vs. 59.07 ± 7.09 μm, t = 8.44, *P*<0.001) and outer retinal thickness (224.47 ± 28.42 μm vs. 183.79 ± 15.98 μm, t = 4.70, *P*<0.001). In contrast, choroidal thickness (282.76 ± 45.83 μm vs. 295.58 ± 39.27 μm, t = -0.79, *P* = 0.44) was slightly decreased in the IERM surgery group as compared to the control group, but the difference was not statistically significant (Table 2; Fig. 2A).

**Table 2:** The retina and choroidal thickness of the idiopathic epiretinal membrane (IERM) surgery group and the control group

Thickness(µm)	IERM surgery group	Control group	t	*P* value
Central foveal	305.47 ± 69.67	204.67 ± 17.40	5.58	<0.001***
Inner retina	85.07 ± 9.26	59.07 ± 7.09	8.44	<0.001***
Outer retina	224.47 ± 28.42	183.79 ± 15.98	4.70	<0.001***
Choroidal	282.76 ± 45.83	295.58 ± 39.27	-0.79	0.44

*P＜0.05, **P＜0.01,  ***P＜0.001

Data are expressed as mean ± standard deviation. Differences between groups were assessed using the independent samples t-test. t, t value of the independent samples t-test. Central foveal, inner retinal, and outer retinal thickness were significantly higher in the IERM surgery group compared with the control group, whereas choroidal thickness showed a slight but nonsignificant decrease

### OCTA measurement

In the OCTA examination, the mean VD of SCP at both 1 mm and 3 mm diameter area was observed to be significantly elevated in the IERM surgery group as compared to the control group (1 mm: 17.37 ± 2.88 vs. 7.70 ± 3.77, t = 7.79, *P*<0.001; 3 mm: 21.03 ± 2.00 vs. 18.39 ± 2.27, t = 3.32, *P* < 0.01). Subfield analysis revealed a statistically significant increase of VD in the IERM surgery group across the nasal and temporal area compared to the control group (nasal: 21.25 ± 1.82 vs. 18.97 ± 3.23, t = 2.32, *P* = 0.03; temporal: 22.30 ± 2.01 vs. 19.66 ± 2.35, t = 3.27, *P* < 0.01). The FAZ area was significantly reduced in the IERM surgery group (0.09 ± 0.10 in IERM vs. 0.23 ± 0.16 in Control, t= -2.86, *P* = 0.01). The blood flow characteristics data are meticulously presented in Table 3; Fig. 2B.

**Table 3:** Percentage vessel density (VD) in the superficial capillary plexus (SCP) and foveal avascular zone area (FAZ) in 3 × 3 mm scan of the idiopathic epiretinal membrane (IERM) surgery group and the control group

	IERM surgery group	Control group	t	*P* value
SCP VD-foveal, %	17.37 ± 2.88	7.70 ± 3.77	7.79	<0.001***
SCP VD-parafoveal, %	21.03 ± 2.00	18.39 ± 2.27	3.32	< 0.01**
SCP VD-para-S, %	21.39 ± 2.47	20.62 ± 2.01	0.91	0.37
SCP VD-para-I, %	21.37 ± 2.43	19.26 ± 3.62	1.85	0.08
SCP VD-para-N, %	21.25 ± 1.82	18.97 ± 3.23	2.32	0.03*
SCP VD-para-T, %	22.30 ± 2.01	19.66 ± 2.35	3.27	< 0.01**
Foveal avascular zone area (mm^2^)	0.09 ± 0.10	0.23 ± 0.16	-2.86	0.01*

*P＜0.05,  **P＜0.01,  ***P＜0.001

Data are presented as mean ± standard deviation. Differences between groups were assessed using the independent samples t-test. t, t value of the independent samples t-test. The percentage VD in the SCP and the FAZ measured using a 3 × 3 mm scan were compared between the IERM surgery group and the control group, and the results of the statistical analyses are reported. The foveal and parafoveal VD in the SCP were significantly higher in the IERM surgery group than in the control group. Subfield analysis further demonstrated a significant increase in VD in the nasal and temporal sectors in the IERM surgery group. In addition, the FAZ area was significantly smaller in the IERM surgery group compared with the control group. All quadrant analyses were performed at an eccentricity of 3°

### Assessment of cone morphology using AO-SLO

**Fig. 3:**
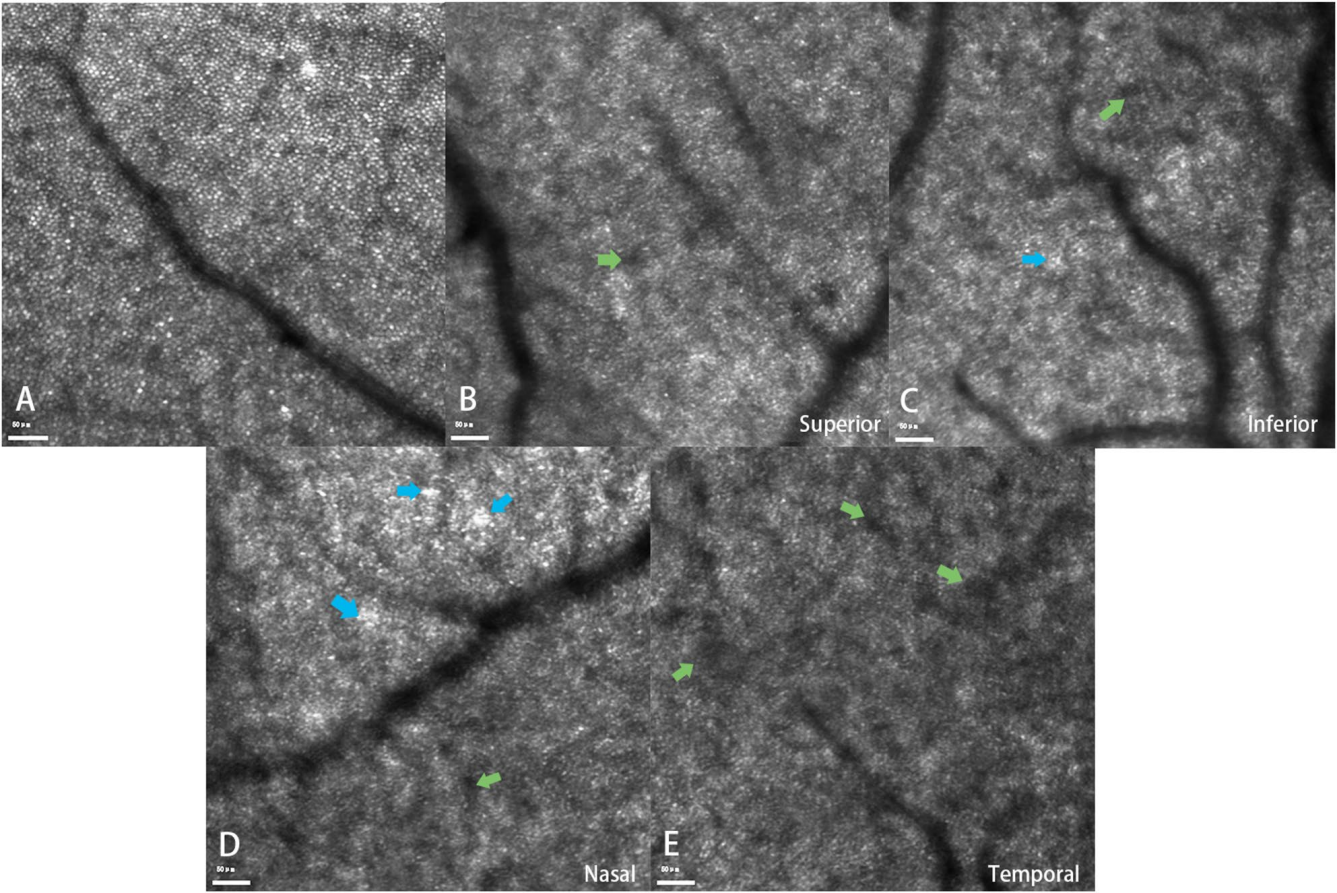
Representative AO-SLO image of a normal healthy eye (retinal field of view: 2.4° × 2.4°), showing cone photoreceptors as regularly arranged circular spots (**A**). Representative AO-SLO images from four quadrants of a postoperative IERM patient (2.4° × 2.4°), demonstrating irregular and clustered cone mosaic patterns. Blue arrows indicate clustered cone photoreceptors, while green arrows highlight patchy hyporeflective areas, suggesting disruption of the normal cone mosaic architecture (**B**–**E**)

All subjects successfully completed the AO-SLO examination. In AO-SLO imaging, normal cone mosaics are typically characterized by a regular arrangement of uniformly bright, circular spots [14, 15]. However, in all 17 eyes of patients following IERM surgery, the cone mosaics exhibited irregular arrangements or clustered patterns **(**Fig. 3B–E**)**. Clustered cone photoreceptors (blue arrows) were frequently observed, replacing the regular hexagonal mosaic seen in healthy eyes. Individual circular spots appeared noticeably darker than those observed in healthy eyes. Moreover, patchy hyporeflective regions (green arrows) were prominent, suggesting localized alterations in cone reflectivity and mosaic integrity **(**Fig. 3**)**. For quantitative cell analysis, compared to the control group, the mean cone density and regularity were significantly reduced in the IERM surgery group (Fig. 2C), while the mean cone dispersion was significantly increased (mean cone densities: t=-4.76; *P* < 0.001; mean cone regularity: t=-5.11, *P* < 0.001; mean cone dispersion: t = 5.56, *P* < 0.001). Similar findings were observed in a subfield analysis at the four quadrants (All *P* < 0.01). Detailed information is presented in Table 4.

**Table 4:** Cone morphology at four quadrants of idiopathic epiretinal membrane (IERM) surgery group and control group

Quadrant	Cone Morphology	IERM surgery group	Control group	t	*P* value
Mean Superior	Cone densities Cone dispersion Cone regularity Cone densities	9341.88 ± 3189.39 0.40 ± 0.07 0.89 ± 0.01 8740.07 ± 3373.70	13270.82 ± 2582.68 0.28 ± 0.07 0.92 ± 0.02 11794.88 ± 2810.30	-4.76 5.56 -5.11 -3.44	<0.001*** <0.001*** <0.001*** <0.001***
	Cone dispersion	0.41 ± 0.11	0.30 ± 0.08	4.01	<0.001***
	Cone regularity	0.89 ± 0.02	0.92 ± 0.03	-3.04	<0.01**
Inferior	Cone densities	9106.92 ± 3395.93	12674.08 ± 3634.00	-3.39	<0.001***
	Cone dispersion	0.40 ± 0.08	0.29 ± 0.09	4.86	<0.001***
	Cone regularity	0.89 ± 0.02	0.92 ± 0.03	-4.45	<0.001***
Nasal	Cone densities	9682.71 ± 3838.41	13809.08 ± 3703.30	-3.73	<0.001***
	Cone dispersion	0.38 ± 0.09	0.28 ± 0.09	3.85	<0.001***
	Cone regularity	0.89 ± 0.03	0.92 ± 0.03	-3.98	<0.001***
Temporal	Cone densities	9837.81 ± 4816.82	14805.23 ± 3360.32	-3.82	<0.001***
	Cone dispersion	0.41 ± 0.11	0.27 ± 0.08	5.23	<0.001***
	Cone regularity	0.87 ± 0.02	0.93 ± 0.03	-6.24	<0.001***

*P＜0.05,  **P＜0.01,  ***P＜0.001

Data are presented as mean ± standard deviation. Differences between groups were assessed using the independent samples t-test. t, t value of the independent samples t-test. In the IERM surgery group, cone density and regularity were significantly reduced, whereas cone dispersion was significantly increased compared with the control group. All quadrant analyses were performed at an eccentricity of 3°

### Factors associated with cone morphology

**Fig. 4:**
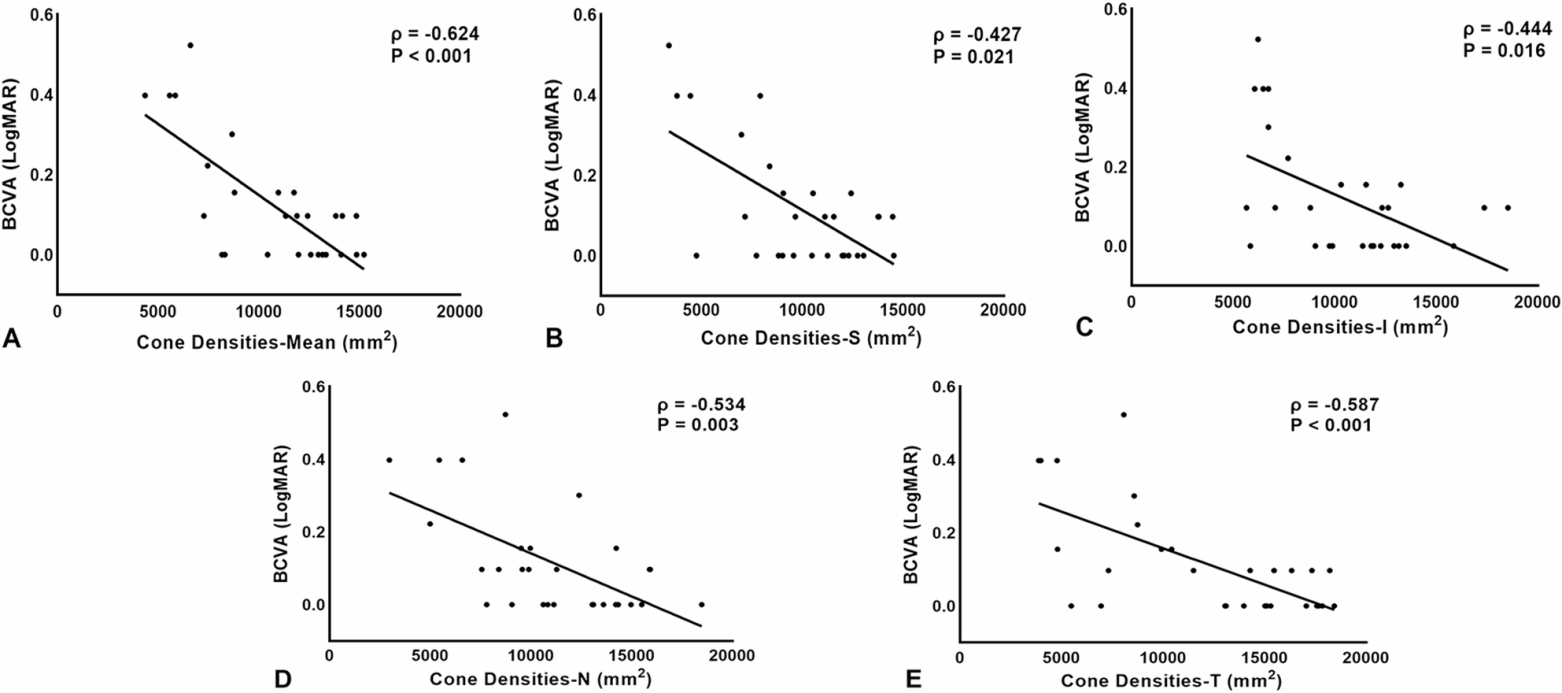
Relationship between the cone densities of the four quadrants and logMAR BCVA. Mean cone density and cone densities in all quadrants showed a negative correlation with logMAR BCVA (**A**-**E**). Spearman correlation coefficients (ρ) and P values are presented in the corresponding graphs. Statistical significance was defined as *P* < 0.05

To evaluate which factors are associated with cone morphology post-IERM surgery, this study conducted a correlation analysis between the cone density at all quadrants and BCVA, as well as the OCT and OCTA findings. A significant negative correlation was identified between the cone densities at all quadrants and BCVA, expressed as logMAR (all *P*<0.05,Table 4; Fig. 4). Additionally, the correlation analysis between cone density and retinal microstructure demonstrated a statistical negative correlation between the mean cone density and the inner retinal thickness(ρ=-0.38, *P* = 0.04). Specifically, densities in the inferior quadrants exhibited a significantly negative correlation with the thickness of the inner retina. However, no statistically significant correlation was observed with the central and outer retinal thickness. In terms of the relationship between cone morphology and OCTA characteristics, a significantly negatively correlation was found between the mean densities of cones and the VD of SCP in the fovea (ρ=-0.39, *P* = 0.04). However, subfield correlation analysis did not find a significant association between cone densities and the VD of the SCP (Table 5).

**Table 5:** Correlation between cone densities in different quadrants and OCT, OCTA characteristics, and visual function in the idiopathic epiretinal membrane (IERM) surgery group and control group

Characteristics	Cone Densities-Mean		Cone Densities-S		Cone Densities-I		Cone Densities-*N*		Cone Densities-T	
	ρ	*P* value	ρ	*P* value	ρ	*P* value	ρ	*P* value	ρ	*P* value
BCVA (Log MAR)	-0.62	<0.001***	-0.43	0.02*	-0.44	0.02*	-0.53	< 0.01**	-0.59	<0.001***
MS (dB)	-0.20	0.30	-0.23	0.23	-0.07	0.721	-0.34	0.074	-0.11	0.56
Central macular thickness (µm)	-0.31	0.10	-0.20	0.32	-0.19	0.34	-0.01	0.98	-0.29	0.15
Inner retinal thickness (µm)	-0.38	0.04*	-0.06	0.79	-0.42	0.03*	-0.16	0.44	-0.30	0.13
Outer retinal thickness (µm)	-0.06	0.75	-0.05	0.79	-0.16	0.41	0.17	0.40	-0.01	0.96
Fovea avascular zone area (mm^2^)	0.10	0.59	-0.03	0.87	0.27	0.16	-0.08	0.69	0.22	0.26
SCP VD-fovea, %	-0.39	0.04*	-0.25	0.19	-0.35	0.06	-0.26	0.17	-0.39	0.04*
SCP VD-parafovea, %	-0.17	0.39	-0.08	0.67	-0.06	0.74	-0.11	0.57	-0.21	0.27
SCP VD-para-S, %	-0.04	0.85	-0.04	0.83	**—**	**—**	**—**	**—**	**—**	**—**
SCP VD-para-I, %	-0.07	0.71	**—**	**—**	-0.07	0.73	**—**	**—**	**—**	**—**
SCP VD-para-N, %	-0.13	0.49	**—**	**—**	**—**	**—**	-0.15	0.45	**—**	**—**
SCP VD-para-T, %	-0.19	0.32	**—**	**—**	**—**	**—**	**—**	**—**	-0.23	0.23

*P＜0.05,  **P＜0.01,  ***P＜0.001

Correlation analysis using Pearson correlation analysis

## Discussion

Idiopathic IERM involves fibrotic proliferation of the posterior vitreous, forming a contractile membrane that exerts centripetal traction on the retina, potentially disrupting photoreceptor alignment and the ellipsoid zone (EZ) layer, detectable on OCT [16]. The integrity of the EZ layer correlates with baseline visual acuity, and its recovery post-treatment is linked to improved visual outcomes [17–20]. Thus, maintaining photoreceptor health in the fovea is crucial for visual recovery after IERM surgery, emphasizing the need for thorough evaluation and monitoring. In this study, we employed AO-SLO to assess cone cell morphological changes following IERM surgery, correlating cone density with visual function, retinal structure, and blood flow characteristics. Our findings revealed that, compared to healthy controls, post-surgery patients showed reduced cone density, irregularity, and increased dispersion. Cone density was significantly associated with visual acuity, the integrity of inner retinal layers, and superficial capillary density within 1 mm of the macular region. These results offer new insights into how changes in cone cell density, regularity, and dispersion influence visual function recovery after IERM surgery.

In this study, we observed a significant reduction in BCVA and MS among patients who underwent vitrectomy for the treatment of an IERM when compared to the healthy control eyes. Postoperative OCT assessments revealed that even after surgery for IERM, the impact of preoperative centripetal traction on the macula persists, make it difficult for central macular thickness, inner retinal thickness, and outer retinal thickness to return to levels comparable to those of a normal cohort. OCTA examination showed a significantly higher vessel density in the superficial capillary plexus in the IERM surgery group compared with the control group, which may reflect recovery of vascular architecture. Interestingly, this enhanced VD exceeds that of healthy eyes, a result that contrasts with prior researches [21–23]. One plausible explanation for this discrepancy is that the traction exerted by the ERM induces centripetal displacement of the SCP, and subsequent postoperative structural recovery of these capillaries, influenced by this displacement, results in an elevated VD that manifests even high than normal at more than 6 months postoperatively [24]. Furthermore, OCTA measurements corroborated earlier findings of a significantly reduced FAZ size in post-surgery patients, likely a sequel to the centripetal traction previously exerted by the IERM [25, 26]. 

Utilizing AO-SLO, we observed a statistically significant decrease in cone densities and regularity at all quadrants, concurrent with an increase of cone dispersion. Under normal conditions, photoreceptor cells are arranged in a hexagonal pattern, creating a uniform mosaic visible with AO-SLO, but the context of certain retinal pathologies, cellular damage can lead to morphological abnormalities, characterized by irregular cell shapes and decreased cell density [27–32]. This study substantiates the findings of Ooto S et al., who proposed that centripetal traction exerted by the IERM leads to reduced cell density and disrupted cell arrangement [33]. Furthermore, previous study has demonstrated that following surgical intervention, the morphology and function of cone exhibit partial restoration, rendering cone density a reliable surrogate marker for visual function. Based on previous research findings, this study found a significant correlation between cone density and BCVA following IERM surgery, including both the average cone cell density and the densities in each quadrant. This indicates that cone density can reflect changes in visual acuity following IERM surgery. However, the extent of cone loss may not perfectly align with MS [34, 35]. Further investigation with a larger sample size is needed to establish the relationship with retinal sensitivity.

In correlation analysis between cone density and retinal structure, we observed negative correlation between inner retinal thickness and average cone density, indicating that as the thickness of the inner retina increases, the density of cone cells decreases. Numerous previous studies have demonstrated that IERM impairs the inner retinal layer in a traction force-dependent manner, leading to the thickening of the inner retinal layers, a decline in visual function, and distortion of vision [36–40]. Additionally, the thickness of the inner retina has been clearly associated with visual acuity following IERM surgery. Based on the research findings and our study’s results, it is plausible to suggest that the tractional force of IERM not only damages the structure of the inner retina but may also affect the structure and arrangement of cone cells through some form of transduction mechanism [41]. Furthermore, our analysis of cone density and macular vascular structure revealed a correlation between the VD of the SCP in the fovea and average cone density. However, in subfield analysis, no significant correlation was observed between cone density and the VD of the SCP. Although SCPs are not directly histologically linked to photoreceptors, studies have indicated that an increase in foveal VD of SCP is in correlation to the contraction from the periphery to the center of the membrane [24, 42]. Consequently, the greater the tractional force exerted by the IERM, the lower VD of the SCP, leading to greater structural disruption of the cone cells. Additionally, membranes with greater tractional force require more forceful dissection during surgery, potentially causing more damage to the Müller cells and the nerve fiber layer. Whether this could indirectly lead to a decrease in cone density warrants further investigation and validation.

Multiple comparisons should be considered when interpreting the correlation analyses, which were performed in an exploratory manner without formal correction to avoid inflating type II error. The primary findings, particularly the strong correlations between cone density and BCVA (*P* < 0.001), showed large effect sizes and appear robust, whereas correlations with borderline significance should be interpreted cautiously. Future studies with larger sample sizes and pre-specified hypotheses are needed to confirm these results and allow appropriate correction for multiple comparisons.

This study has several limitations. First, the relatively small sample size increases the risk of both false-negative and false-positive findings. In addition, the absence of eyes with untreated IERM limits our ability to directly evaluate cone photoreceptor alterations attributable to the disease itself; investigating untreated IERM eyes may provide further insight into cone morphological changes associated with IERM. Second, as a cross-sectional study, longitudinal data were not available, which restricts a comprehensive assessment of cone photoreceptor recovery following macular surgery. Third, the current spatial resolution of AO-SLO remains insufficient for detailed visualization of foveal cone photoreceptors. Further technological advances in imaging are required to enable more precise and direct observation of these structures. Fourth, future studies incorporating a broader range of functional and morphological parameters will be necessary to better elucidate the relationships between imaging findings and clinical phenotypes. Fifth, surgical intervention itself may influence cone photoreceptor imaging outcomes. Future investigations should specifically address the effects of surgical procedures on cone imaging. In addition, the severity of IERM may affect the measurements, highlighting the need for more refined stratification of disease stage in subsequent studies.

In conclusion, this study investigated the morphological alterations of cone cells post-IERM surgery and elucidated the correlation between these alterations and visual function. These findings provide new insights into the complex mechanisms between cone cell alterations and visual function recovery in patients following the surgery. This research highlights the importance of further exploration into the relationship between cone cell changes and visual function in IERM patients, as well as the potential for utilizing cone cell morphology as a prognostic indicator for visual outcomes in IERM patients in the future.

## Data Availability

The datasets used or analysed during the current study are available from the corresponding author on reasonable request.

## Abbreviations

IERM: Idiopathic epiretinal membrane
AO-SLO: Adaptive optics scanning light ophthalmoscopy
OCT: Optical coherence tomography
OCTA: Optical coherence tomography angiography
BCVA: Best-corrected visual acuity
AL: Axial length
RT: Retinal thickness
VD: Vascular densities
SCP: Superficial capillary plexuse
FAZ: Foveal avascular zone
MS: Mean retinal sensitivity
SE: Spherical equivalent
EZ: Ellipsoid zone
